# Associations between polymorphisms in leptin and leptin receptor genes and colorectal cancer survival

**DOI:** 10.20892/j.issn.2095-3941.2022.0635

**Published:** 2023-06-06

**Authors:** Meizhi Du, Yu Wang, Jillian Vallis, Matin Shariati, Patrick S. Parfrey, John R. Mclaughlin, Peizhong Peter Wang, Yun Zhu

**Affiliations:** 1 Department of Epidemiology and Biostatistics, School of Public Health, Tianjin Medical University, Tianjin 300070, China; 2 Division of Community Health and Humanities, Faculty of Medicine, Memorial University of Newfoundland, St. John’s A1B 3V6, Canada; 3 Clinical Epidemiology Unit, Faculty of Medicine, Memorial University of Newfoundland, St. John’s A1B 3V6, Canada; 4 Dalla Lana School of Public Health, University of Toronto, Toronto M5T 3M7, Canada; 5 Beatrice Hunter Cancer Institute, Halifax B3H 4R2, Canada; 6 Centre for New Immigrant Wellbeing, Markham L3R 9V1, Canada

**Keywords:** *LEP*, *LEPR*, polymorphism, gene-environment interaction, colorectal cancer survival

## Abstract

**Objective:**

Leptin (LEP) is an obesity-associated adipokine associated with tumor cell growth. We examined the relevance of genetic variants of *LEP* and leptin receptor (*LEPR*) to colorectal cancer (CRC) survival by using data from the Newfoundland Familial Colorectal Cancer Study.

**Methods:**

A total of 532 patients newly diagnosed with CRC between 1997 and 2003 were followed up until April 2010. Data on their demographics and lifestyles were collected *via* questionnaires. Genotyping of blood samples was performed with the Illumina Human Omni-Quad Bead chip. Multivariable Cox models were used to assess the relationships of 35 tag single-nucleotide polymorphisms (SNPs) in *LEP* and *LEPR* with overall survival (OS), disease-free survival (DFS), and CRC-specific survival.

**Results:**

At the gene level, *LEP* was associated with DFS (*P* = 0.017), and *LEPR* was associated with both DFS (*P* = 0.021) and CRC-specific survival (*P* = 0.013) in patients with CRC. In single-SNP analysis, *LEP* rs11763517, *LEPR* rs9436301, and *LEPR* rs7602 were associated with DFS after adjustment for multiple testing. The *LEPR* haplotypes G-C-T (rs7534511-rs9436301-rs1887285) and A-A-G (rs7602-rs970467-rs9436748) were associated with prolonged OS among patients with CRC overall (G-C-T: HR, 0.63; 95% CI, 0.43–0.93; A-A-G: HR, 0.59; 95% CI, 0.38–0.91) and those diagnosed with colon cancer (G-C-T: HR, 0.54; 95% CI, 0.34–0.86; A-A-G: HR, 0.49; 95% CI, 0.29–0.83). Similar results were observed for DFS. Moreover, significant interactions were found among *LEPR* rs7602 (A *vs.* G), *LEPR* rs1171278 (T *vs*. C), red meat intake, and BMI status: the associations between these variants and prolonged DFS were limited to patients with below-median red meat consumption and body mass index (BMI) < 25 kg/m^2^.

**Conclusions:**

Polymorphic variations in the *LEP* and *LEPR* genes were associated with survival of patients after CRC diagnosis. The *LEP*/*LEPR*-CRC survival association was modified by participants’ red meat intake and BMI.

## Introduction

Colorectal cancer (CRC) is the third most common malignancy and the second most deadly cancer worldwide^1^. Obesity is a complex epidemic disease involving metabolic alterations that can trigger various other diseases, including CRC. The mechanisms through which obesity is associated with increased CRC risk may be explained by factors such as hyperinsulinemia, oxidative stress, inflammation, and alterations in adipokine concentrations^2^. Leptin (LEP), the most abundant adipokine, has key roles in suppressing appetite and food intake, thus regulating energy homeostasis and body weight^3^. High levels of serum LEP have been detected in obese people, who have LEP resistance and thus do not benefit from LEP’s anorexigenic effects^4^.

LEP has been associated with elevated risk of developing CRC, as supported by *in vitro*, *in vivo*, and large epidemiological studies^5^. By binding its receptor, LEPR, LEP stimulates cell proliferation, inhibits apoptosis, and promotes angioneogenesis at various levels *via* several signaling pathways (e.g., JAK2/STAT3, PI3K/AKT, and MAPK/ERK)^5–7^. LEP also has proinflammatory properties that promote colon carcinogenesis^5^.

Polymorphisms in the *LEP* and *LEPR* genes are increasingly being studied in conjunction with LEP levels, to provide insight into their roles in obesity-mediated cancers, although data directly linking *LEP* and *LEPR* genetic variations to CRC are limited. Several single-nucleotide polymorphisms (SNPs) have been implicated in CRC pathogenesis; notably, the *LEP* rs2167270 (GG) and *LEPR* rs12037879 (GA/GG) genotypes are associated with elevated risk of CRC^8–11^. *LEP* and *LEPR* have also been suggested to be involved in survival after CRC diagnosis. Research has suggested that *LEP* mRNA expression levels are upregulated in colon cancer tissue and are associated with poor prognosis in patients with colon cancer^12,13^. Similarly, *LEPR* is overexpressed in primary CRC relative to normal colonic mucosa; intriguingly, however, *LEPR* positive tumors have been associated with superior overall survival (OS) in patients^14,15^. Nevertheless, no study to date has examined the polymorphic profiles of the *LEP* and *LEPR* genes in relation to CRC survival. Current understanding of the link between polymorphic variants and CRC survival is based on contradictory and inconclusive data suggesting a potential association of *LEP/LEPR* genetics with cancer risk.

Beyond the inherited genetic background, environmental components and their interactions interfere with CRC initiation and progression. However, the relationships of *LEP* and *LEPR* with modifiable lifestyle factors [e.g., intake of red meat and body mass index (BMI)], particularly the extent to which lifestyle factors may modulate this genetic risk, remains unknown. Thus, potential gene-environment interactions require further investigation to provide new insights that may lead to novel therapeutic targets and prevention strategies.

We therefore analyzed genetic variation in the *LEP* and *LEPR* genes in relation to CRC survival through a tag SNP approach to probe common genetic variations and construct haplotype blocks in the 2 genes. We further examined whether these associations might be modified by behavioral risk factors.

## Materials and methods

### Study population

The data were drawn from the Newfoundland and Ontario Familial Colorectal Cancer Study, a population-based cohort study investigating environmental and genetic components in CRC. The study methods and detailed rationale have been described before^16–18^. In brief, the participants were newly diagnosed with CRC between 1997 and 2003 and were 20–75 years of age at the time of diagnosis. A total of 532 patients with CRC (202 women and 330 men) residing in the provinces of Newfoundland and Labrador were identified through the Newfoundland Familial Colorectal Cancer Registry. The Health Research Ethics Authority of Memorial University of Newfoundland approved the study (Approval No. 40001511). Informed consent was obtained from each patient before participation.

### Diet assessment and baseline information collection

At baseline, all patients with CRC completed a detailed family history questionnaire, a personal History Questionnaire, and a food frequency questionnaire, in which information on demographics, lifestyles, and dietary habits was gathered. All questionnaires were self-administered with a reference period of 1 year before diagnosis, to capture pre-diagnosis information. The median time from the date of diagnosis to questionnaire completion was 1.8 years. The dietary questionnaire was adapted from the Hawaii semi-quantitative food frequency questionnaire and was validated in the Newfoundland population^19^. BMI was defined as the weight in kilograms divided by the square of the height in meters. Information on weight and height was self-reported, and obtained from the PHQ with the following questions: “About how tall are you, without your shoes on?” and “How much did you weigh about 1 year before your recent cancer diagnosis?” Self-reported measures of weight and height are believed to be valid alternatives for determining weight status^20^.

### Study outcomes

All study participants were followed, and death, cancer recurrence, and metastasis from the date of diagnosis until April 2010 were recorded. The endpoints for this study included OS, defined as the time from CRC diagnosis to death due to any cause; disease-free survival (DFS), defined as the time from CRC diagnosis to death due to any cause, CRC recurrence, or metastasis, whichever came first; and CRC-specific survival, measured from the date of diagnosis to the date of death due to CRC.

### SNP selection and genotyping

Genotyping of peripheral blood samples from participants was performed at Centrillion Biosciences (USA) with the Illumina Human Omni-Quad Bead chip, which contains approximately 1.1 million SNPs. Additionally, 200 duplicates were genotyped with the Affymetrix Axiom myDesign GW Array Plate, which contains 1.3 million probes. SNPs with genotype concordance < 97% between platforms were excluded from this analysis.

Data cleaning and quality control filtering were conducted with Plink v1.07. Tag SNPs capturing common genetic variations in the candidate genes were selected with Plink v1.07 according to the following criteria: minor allele frequency > 5%; HWE *P* > 0.001; and linkage disequilibrium (LD) pruning with the Plink option “-indep-pairwise 50 2 0.8.” This process identified 3 SNPs for *LEP* and 29 SNPs for *LEPR*. We additionally included 3 high interest SNPs reported in previous studies (*LEPR* rs1137101, *LEPR* rs1137100, and *LEPR* rs1805096).

Specific information on MSI testing and mutation detection on BRAF V600E in tumor DNA has been reported previously^18^. MSI status in CRCs was determined by DNA testing with 5–10 microsatellite markers. An allele-specific polymerase chain reaction technique was used to detect mutant alleles in the *BRAF* gene^18^.

### Statistical analysis

The log rank test was used to compare the survival distributions of the baseline characteristics. We tested the overall association of genes with principal component (PC) analysis. Single-SNP analysis and haplotype analysis were used to further explore variants in the *LEP* and *LEPR* genes in relation to CRC survival. The PCs were modeled with Cox proportional hazards regression, by using at least an 80% explained-variance threshold for determining the number of PCs to retain in the models. With the likelihood ratio test, we calculated the *P*-values for global associations between genes and disease outcomes by comparing 2 models with and without selected PCs, with degrees of freedom equal to the number of PCs. All analyses were stratified by anatomical site (i.e., the colon and rectum).

The data were further explored in a single-SNP analysis, with an additive model by Cox regression analysis. The decision for variable inclusion in the final model was based on statistical significance, according to stepwise regression with a *P*-value threshold of 0.05. The covariates eventually included in the model were age at diagnosis, gender, race, stage at diagnosis, household income, reported screening procedure, marital status, family history, smoking status, alcohol consumption status, folate intake, MSI status, and BRAF mutation status. Hazard ratios (HRs) and 95% confidence intervals (CIs) were applied to estimate the relationships of individual SNPs with OS, DFS, and CRC-specific survival. To control for type I error inflation, a multiple comparison adjustment specifically created for correlated tests due to LD was used^21^.

LD plots were generated with Haploview version 4.2 to identify haplotype blocks. PHASE version 2.1 was used to estimate the haplotypes in each block. The relationship between haplotype and survival in patients with CRC was assessed with Cox regression modeling, with reference to the most common haplotype. Bonferroni correction for multiple testing was performed for 37 haplotypes, thus yielding an adjusted *P*-value of 0.0014. A global *P* value for each haplotype block was obtained with a likelihood ratio test. Gene-environment interactions were estimated with stratified analyses (by intake of red meat and BMI) and the Wald method through introduction of a multiplicative interaction term into the model and assessment of its significance. Analyses were performed in SAS software version 9.4 (SAS Institute, Cary, NC, USA) and GraphPad 8.0.2. All tests were 2-sided.

## Results

### Patient characteristics and clinical predictors

The study population consisted of 330 men and 202 women. The mean age of the study population was 60.1 ± 9.2 years; 72.4% of the participants had a history of smoking; 96.9% of the participants were white; and 11.5% reported a bowel screening history (**Table 1**). Information on MSI status was obtained in 504 patients, of whom 11.5% were classified as MSI-H, and 88.5% were classified as MSS/MSI-L. In this study, almost all (96%) patients received surgery, and 21% underwent radiation or chemotherapy. At the end of the follow-up, 183 of the 532 patients had died. Most deaths (90.4%) were due to CRC. In the log-rank univariate analysis, male, advanced stage at diagnosis (IV), non-white race, chemoradiotherapy, consumption of > 3 servings of red meat per week, and MSS/MSI-L tumors were significantly associated with shorter OS, whereas bowel screening procedure, smoking status, alcohol consumption status, surgery, and *BRAF* mutation status were not associated with OS.

**Table 1:** Demographical and clinicopathological characteristics of patients in the Newfoundland Familial Colorectal Cancer Study

Characteristic	No. of patients (%)	No. of deaths (%)	MST (Y)	*P*-value
Age at diagnosis (y)^a^	60.1 ± 9.2	60.7 ± 9.8	–	–
Gender				0.005
Female	202 (37.97)	56 (30.60)	6.5	
Male	330 (62.03)	127 (69.40)	6.3	
Race				0.009
White	440 (96.92)	133 (94.33)	6.4	
Other	14 (3.08)	8 (5.67)	4.7	
Stage at diagnosis				< 0.001
I	94 (17.67)	18 (9.84)	6.4	
II	209 (39.29)	58 (31.69)	6.6	
III	178 (33.46)	65 (35.52)	6.4	
IV	51 (9.59)	42 (22.95)	3.9	
Reported screening procedure				0.059
Yes	52 (11.45)	10 (7.09)	6.6	
No	402 (88.55)	131 (92.91)	6.4	
Average alcoholic drinks per week				0.062
0	170 (39.44)	46 (34.59)	6.5	
≤ 7	138 (32.02)	43 (32.33)	6.4	
8–14	74 (17.17)	23 (17.29)	6.4	
> 14	49 (11.37)	21 (15.79)	5.9	
BMI (kg/m^2^)				0.097
< 18.4	8 (1. 60)	6 (3.57)	4.7	
18.5–24.9	138 (27.60)	41 (24.40)	6.4	
25.0–29.9	205 (41.00)	74 (44.05)	6.4	
≥ 30.4	149 (29.80)	47 (27.98)	6.3	
Smoking				0.133
Yes	375 (72.39)	136 (77.71)	6.4	
No	143 (27.61)	39 (22.29)	6.4	
Red meat intake (servings/week)				0.048
< 2	84 (16.47)	25 (14.71)	6.7	
2–3	257 (50.39)	86 (50.59)	6.4	
4–5	83 (16.28)	34 (20.00)	6.2	
> 5	86 (16.86)	25 (14.71)	6.3	
MSI				< 0.001
MSS/MSI-L	446 (88.49)	168 (96.55)	6.3	
MSI/H	58 (11.51)	6 (3.45)	6.7	
BRAF mutation status				0.370
Wild type	433 (89.83)	153 (91.07)	6.4	
BRAF V600E mutant	49 (10.17)	15 (8.93)	6.3	
Surgery				0.118
Yes	483 (96.02)	157 (94.58)	6.4	
No	20 (3.98)	14 (5.42)	5.6	
Chemoradiotherapy				0.036
Yes	107 (20.66)	44 (25.14)	6.0	
No	411 (79.34)	131 (74.86)	6.4	

BMI, body mass index; MSI, microsatellite instability; MST, median overall survival time; MSI-H, microsatellite instability-high; MSS/MSI-L, microsatellite stable/microsatellite instability-low. ^a^Continuous variables are presented as mean ± s.d. (standard deviation).

### Association of LEP and LEPR with survival in patients with CRC

To evaluate the overall gene-level association between *LEP* or *LEPR* and CRC survival, we performed PC analysis with OS, DFS, and CRC-specific survival as the endpoints (**Table 2**). At the gene level, *LEP* was significantly associated with DFS (*LEP*, global *P* = 0.017), and *LEPR* was associated with both DFS (global *P* = 0.021) and CRC-specific survival (global *P* = 0.013), both overall and stratified by colorectal subsite. However, we did not observe any meaningful relationship between *LEP* or *LEPR* and OS.

**Table 2:** Associations of *LEP* and *LEPR* genes with colorectal cancer overall survival, disease-free survival (*n* = 532), and CRC-specific survival (*n* = 459)

	Overall survival HR (95% CI)^a^			Disease-free survival HR (95% CI)^a^			CRC-specific survival HR (95% CI)^a^		
	All CRC	Colon cancer	Rectal cancer	All CRC	Colon cancer	Rectal cancer	All CRC	Colon cancer	Rectal cancer
***LEP***									
PC1	0.92 (0.77–1.09)	0.95 (0.77–1.18)	0.86 (0.63–1.18)	0.82 (0.69–0.96)	0.84 (0.69–1.03)	0.76 (0.56–1.03)	0.92 (0.69–1.22)	0.85 (0.59–1.23)	1.00 (0.63–1.60)
Global *P*^b^	0.340	0.715	0.664	0.017	0.100	0.079	0.194	0.382	1.000
***LEPR***									
PC1	1.05 (0.88–1.25)	1.10 (0.87–1.39)	1.00 (0.75–1.33)	0.98 (0.83–1.15)	0.93 (0.75–1.16)	0.98 (0.74–1.30)	0.87 (0.67–1.13)	0.61 (0.40–0.94)	0.87 (0.56–1.37)
PC2	0.80 (0.67–0.97)	0.78 (0.62–0.98)	0.80 (0.54–1.19)	0.72 (0.60–0.86)	0.74 (0.60–0.93)	0.60 (0.48–1.02)	0.61 (0.44–0.84)	0.51 (0.33–0.78)	0.50 (0.24–1.04)
PC3	0.99 (0.84–1.17)	0.87 (0.71–1.08)	1.25 (0.89–1.74)	1.09 (0.92–1.28)	1.01 (0.82–1.25)	1.30 (0.95–1.79)	0.97 (0.74–1.28)	0.62 (0.40–0.94)	1.92 (1.20–3.07)
PC4	1.14 (0.97–1.34)	1.21 (1.00–1.47)	1.12 (0.82–1.53)	1.15 (0.99–1.33)	1.23 (1.02–1.50)	1.03 (0.79–1.36)	1.32 (1.04–1.68)	1.51 (1.03–2.20)	1.40 (0.92–2.14)
PC5	0.92 (0.78–1.10)	0.95 (0.76–1.17)	0.90 (0.63–1.28)	1.01 (0.86–1.18)	1.03 (0.84–1.27)	0.95 (0.70–1.30)	1.07 (0.81–1.40)	1.12 (0.75–1.68)	1.36 (0.78–2.38)
PC6	0.96 (0.81–1.14)	0.96 (0.77–1.19)	0.91 (0.66–1.25)	0.99 (0.84–1.17)	1.01 (0.82–1.24)	0.94 (0.71–1.25)	0.87 (0.66–1.17)	0.81 (0.53–1.22)	1.02 (0.60–1.71)
PC7	1.06 (0.89–1.26)	1.11 (0.87–1.42)	1.06 (0.80–1.40)	1.10 (0.93–1.29)	1.05 (0.84–1.31)	1.19 (0.91–1.57)	1.26 (0.93–1.72)	1.55 (0.97–2.47)	2.14 (1.18–3.87)
PC8	0.91 (0.76–1.07)	0.84 (0.69–1.03)	1.09 (0.72–1.63)	0.96 (0.81–1.14)	0.91 (0.74–1.11)	1.04 (0.73–1.49)	1.02 (0.78–1.34)	0.92 (0.66–1.29)	0.72 (0.31–1.72)
Global *P*^b^	0.251	0.103	0.832	0.021	0.143	0.344	0.013	0.002	0.020

CRC, colorectal cancer; HR, hazard ratio; PC, principal component. ^a^Cox proportional hazard model adjusted for age at diagnosis, gender, race, stage at diagnosis, household income, reported screening procedure, marital status, family history, smoking status, alcohol consumption status, folate intake, MSI status, and BRAF mutation status, where applicable. ^b^Global *P* for association is from a likelihood ratio test, with degrees of freedom equal to the number of PCs.

**Figure 1:**
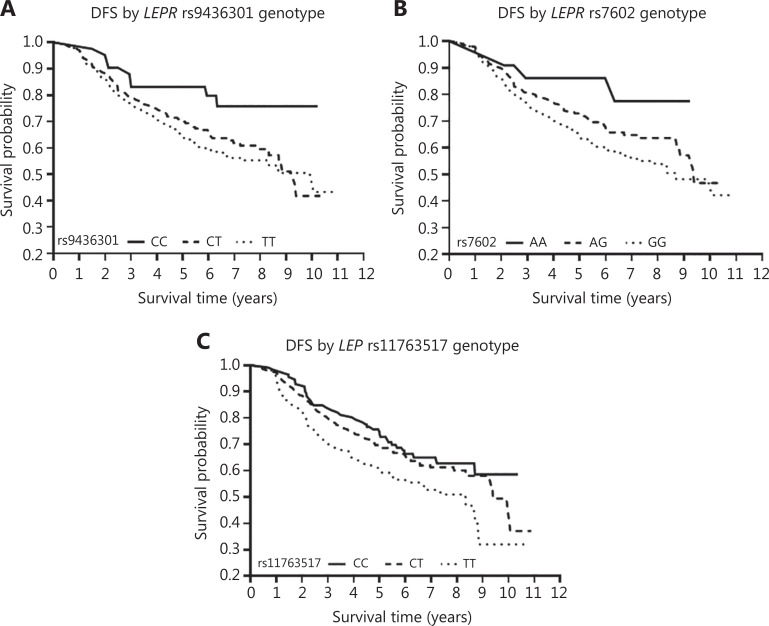
(A) Disease-free survival curves for patients with the *LEPR* rs9436301 genotype. (B) Disease-free survival curves for patients with the *LEPR* rs7602 genotype. (C) Disease-free survival curves for patients with the *LEP* rs11763517 genotype.

The relationships between individual SNPs within each gene and CRC survival were then evaluated with additive models (**Supplementary Tables S1 and S2**). Evaluation of 3 SNPs in *LEP* and 32 SNP in *LEPR* revealed 2 SNPs significantly associated with OS, 6 SNPs significantly associated with DFS, and 8 SNPs associated with CRC-specific survival. However, after adjustment for multiple testing, only *LEP* rs11763517 (*P*_unadjusted_ = 0.001; *P*_adjusted_ = 0.015), *LEPR* rs9436301 (*P*_unadjusted_ = 0.000; *P*_adjusted_ = 0.010), and *LEPR* rs7602 (*P*_unadjusted_ = 0.000; *P*_adjusted_ = 0.008) were associated with the DFS of CRC. Specifically, for *LEP* rs11763517, the C-allele was associated with longer DFS than the T-allele (HR, 0.64; 95% CI, 0.50–0.83) (**Figure 1**); a similar protective effect of the C allele on DFS was found for *LEPR* rs9436301 (HR, 0.60; 95% CI, 0.45–0.79). For *LEPR* rs7602, the A-allele was associated with longer DFS than the G-allele (HR, 0.55; 95% CI, 0.39–0.76). No significant relationship was observed between any SNPs in *LEP* or *LEPR* and overall or CRC-specific survival, after adjustment for multiple comparisons. When we repeated the analyses in patients with CRC diagnosed at a later stage (i.e., stage III/IV), we observed similar results for DFS (*LEP* rs11763517, HR, 0.63; 95% CI, 0.44–0.91; *LEPR* rs7602, HR, 0.39; 95% CI, 0.24–0.63; and *LEPR* rs9436301, HR, 0.55; 95% CI, 0.37–0.82). In addition, this association was more pronounced in advanced-stage cancers (stage III/IV) (*LEPR* rs7602 HR, 0.39, 95% CI, 0.24–0.63; *LEPR* rs1171278, HR, 0.40, 95% CI, 0.24–0.68) than in those detected at an early stage (stage I/II) (*LEPR* rs7602, HR, 0.82, 95% CI, 0.51–1.31; *LEPR* rs1171278, HR, 0.83, 95% CI, 0.49–1.40) (**Supplementary Tables S3 and S4**). Furthermore, analysis stratified by MSI status indicated similar patterns of association for MSI-H and MSS/MSI-L tumors (data not shown).

### Haplotypes and survival of patients with CRC

Haplotype analysis assessed 3 SNP sites of *LEP* and 32 SNP sites of *LEPR* for LD (**Supplementary Figures S1 and S2**). We identified a total of 10 haplotype blocks of *LEP* and *LEPR*, and ignored combinations with frequencies less than 0.01 in the analysis (**Table 3**). For the *LERP* gene, the haplotype G-C-T in LD block 2 (rs7534511-rs9436301-rs1887285) was associated with longer OS among patients with CRC overall (HR, 0.63; 95% CI, 0.43–0.93) and patients with colon cancer (HR, 0.54; 95% CI, 0.34–0.86) than the most common haplotype. The results were similar for DFS (CRC, HR, 0.55; 95% CI, 0.38–0.80; colon cancer, HR, 0.54; 95% CI, 0.34–0.86). The haplotype A-A-G situated in LD block 3 of *LEPR* (rs7602-rs970467-rs9436748) was significantly associated with increased OS (HR, 0.49; 95% CI, 0.29–0.83) and DFS (HR, 0.58; 95% CI, 0.34–0.99) for patients with colon cancer. Notably, SNP *LEPR* rs7602, which had been previously identified as being inversely associated with mortality risk in single SNP analysis, was embedded within this haplotype. However, these associations were rendered nonsignificant after adjustment for multiple testing.

**Table 3:** Associations of *LEP* and *LEPR* gene haplotypes with overall survival, disease-free survival (*n* = 532), and CRC-specific survival (*n* = 459)

Haplotypes	Frequency^b^	Overall survival HR (95% CI)^a^			Disease-free survival HR (95% CI)^a^			CRC-specific survival HR (95% CI)^a^		
		All CRC	Colon cancer	Rectal cancer	All CRC	Colon cancer	Rectal cancer	All CRC	Colon cancer	Rectal cancer
*LEPR*, **block 1**^c^										
CAT	0.441	1.00	1.00	1.00	1.00	1.00	1.00	1.00	1.00	1.00
AGT	0.425	0.97 (0.74–1.28)	1.04 (0.74–1.47)	0.95 (0.58–1.57)	1.03 (0.80–1.34)	1.00 (0.71–1.41)	1.39 (0.87–2.22)	1.03 (0.80–1.34)	1.16 (0.68–1.98)	0.92 (0.48–1.75)
AAC	0.130	0.94 (0.63–1.41)	0.94 (0.58–1.53)	1.14 (0.53–2.49)	0.95 (0.66–1.38)	0.95 (0.60–1.49)	1.15 (0.56–2.37)	0.95 (0.66–1.38)	0.70 (0.32–1.53)	0.61 (0.23–1.64)
Global *P*^d^		0.989	0.992	0.990	0.801	0.989	0.926	0.909	0.909	0.911
*LEPR*, **block 2**^e^										
GTT	0.418	1.00	1.00	1.00	1.00	1.00	1.00	1.00	1.00	1.00
ATT	0.311	0.99 (0.74–1.34)	0.85 (0.58–1.26)	1.33 (0.81–2.18)	1.02 (0.77–1.33)	1.07 (0.74–1.53)	0.90 (0.56–1.46)	1.42 (0.93–2.18)	1.36 (0.75–2.46)	1.34 (0.69–2.61)
GCT	0.180	0.63 (0.43–0.93)	0.54 (0.34–0.86)	0.69 (0.32–1.48)	0.55 (0.38–0.80)	0.54 (0.34–0.86)	0.55 (0.27–1.13)	0.75 (0.40–1.39)	0.70 (0.33–1.50)	0.65 (0.20–2.18)
GCC	0.090	0.87 (0.54–1.40)	0.85 (0.48–1.49)	0.58 (0.21–1.58)	0.69 (0.43–1.11)	0.81 (0.46–1.44)	0.88 (0.35–2.22)	0.56 (0.26–1.18)	0.54 (0.21–1.37)	0.54 (0.11–2.62)
Global *P*^d^		0.266	0.206	0.506	0.009	0.052	0.575	0.063	0.249	0.572
*LEPR*, **block 3**^f^										
GGT	0.422	1.00	1.00	1.00	1.00	1.00	1.00	1.00	1.00	1.00
GGG	0.356	1.03 (0.77–1.37)	0.90 (0.62–1.29)	1.27 (0.78–2.09)	1.12 (0.86–1.46)	1.25 (0.89–1.77)	0.86 (0.53–1.41)	1.42 (0.95–2.13)	1.38 (0.79–2.44)	1.35 (0.71–2.57)
AAG	0.134	0.59 (0.38–0.91)	0.49 (0.29–0.83)	0.81 (0.35–1.90)	0.58 (0.38–0.88)	0.58 (0.34–0.99)	0.52 (0.23–1.21)	0.75 (0.38–1.45)	0.71 (0.32–1.58)	1.60 (0.15–2.24)
AGG	0.081	0.83 (0.51–1.36)	0.79 (0.45–1.38)	0.92 (0.32–2.68)	0.67 (0.41–1.09)	0.72 (0.40–1.28)	0.73 (0.28–1.92)	0.43 (0.17–1.09)	0.47 (0.16–1.39)	0.37 (0.05–2.96)
Global *P*^d^		0.293	0.325	0.929	0.027	0.114	0.800	0.108	0.457	0.630
*LEPR*, **block 4**^g^										
AAAG	0.476	1.00	1.00	1.00	1.00	1.00	1.00	1.00	1.00	1.00
GGAT	0.338	1.03 (0.77–1.39)	0.87 (0.60–1.27)	1.37 (0.81–2.30)	1.03 (0.78–1.35)	1.05 (0.74–1.49)	0.99 (0.59–1.66)	1.23 (0.79–1.90)	1.18 (0.64–2.16)	1.15 (0.59–2.25)
AGGG	0.114	0.69 (0.44–1.08)	0.61 (0.36–1.03)	0.67 (0.29–1.57)	0.55 (0.35–0.84)	0.64 (0.37–1.09)	0.52 (0.24–1.15)	0.64 (0.31–1.34)	0.60 (0.26–1.41)	0.38 (0.09–1.71)
AGAT	0.073	1.27 (0.81–1.98)	1.15 (0.68–1.97)	1.46 (0.61–3.46)	1.31 (0.86–2.00)	1.43 (0.83–2.47)	0.77 (0.34–1.77)	1.48 (0.81–2.70)	1.35 (0.64–2.86)	1.66 (0.56–4.86)
Global *P*^d^		0.041	0.446	0.225	0.038	0.324	0.642	0.329	0.551	0.459
*LEPR*, **block 5**^h^										
TC	0.746	1.00	1.00	1.00	1.00	1.00	1.00	1.00	1.00	1.00
TT	0.159	0.74 (0.51–1.08)	0.66 (0.42–1.04)	0.84 (0.43–1.63)	0.60 (0.42–0.86)	0.65 (0.41–1.02)	0.68 (0.36–1.26)	0.58 (0.31–1.07)	0.62 (0.28–1.33)	0.41 (0.14–1.20)
CT	0.095	1.03 (0.70–1.52)	1.05 (0.65–1.70)	1.11 (0.55–2.25)	1.16 (0.81–1.67)	1.30 (0.81–2.09)	0.81 (0.43–1.56)	1.41 (0.83–2.41)	1.41 (0.72–2.79)	1.63 (0.65–4.06)
Global *P*^d^		0.410	0.282	0.940	0.016	0.104	0.609	0.118	0.378	0.209
*LEPR*, **block 6**^i^										
CTTG	0.479	1.00	1.00	1.00	1.00	1.00	1.00	1.00	1.00	1.00
CCCA	0.289	0.96 (0.71–1.30)	0.85 (0.58–1.25)	1.32 (0.78–2.24)	1.05 (0.79–1.38)	1.11 (0.77–1.60)	0.92 (0.55–1.53)	1.18 (0.76–1.84)	1.09 (0.58–2.03)	0.92 (0.55–1.53)
TTTA	0.148	0.75 (0.51–1.12)	0.67 (0.41–1.09)	0.86 (0.44–1.71)	0.59 (0.40–0.86)	0.62 (0.37–1.01)	0.66 (0.34–1.26)	0.62 (0.32–1.18)	0.57 (0.25–1.27)	0.49 (0.15–1.54)
CTCA	0.071	1.46 (0.94–2.27)	1.46 (0.86–2.48)	1.44 (0.61–3.41)	1.44 (0.94–2.19)	1.66 (0.97–2.83)	0.76 (0.33–1.74)	1.44 (0.80–2.63)	1.32 (0.63–2.78)	1.27 (0.64–2.52)
Global *P*^d^		0.415	0.262	0.852	0.048	0.161	0.946	0.493	0.792	0.712
*LEPR*, **block 7**^j^										
GAAA	0.289	1.00	1.00	1.00	1.00	1.00	1.00	1.00	1.00	1.00
GGAG	0.265	1.04 (0.72–1.49)	1.03 (0.64–1.63)	1.27 (0.69–2.34)	1.25 (0.90–1.74)	1.25 (0.81–1.92)	1.13 (0.63–2.00)	1.32 (0.79–2.22)	1.14 (0.56–2.31)	1.70 (0.75–3.84)
AAAG	0.237	1.27 (0.90–1.79)	1.33 (0.86–2.04)	1.27 (0.68–2.38)	1.39 (1.01–1.92)	1.47 (0.97–2.23)	0.91 (0.51–1.63)	1.29 (0.78–2.14)	1.06 (0.53–2.11)	1.80 (0.74–4.39)
GACA	0.188	0.86 (0.58–1.26)	1.11 (0.69–1.78)	0.83 (0.39–1.78)	1.10 (0.77–1.57)	0.98 (0.62–1.56)	1.44 (0.73–2.84)	1.02 (0.59–1.77)	1.08 (0.55–2.10)	1.15 (0.38–3.49)
GAAG	0.018	0.77 (0.36–1.65)	0.88 (0.34–2.30)	0.96 (0.25–3.75)	0.97 (0.45–2.07)	1.04 (0.38–2.81)	0.91 (0.25–3.41)	1.33 (0.31–5.73)	1.02 (0.12–8.47)	3.58 (0.40–32.19)
Global *P*^d^		0.567	0.919	0.937	0.511	0.638	0.946	0.951	1.000	0.843
*LEPR*, **block 8**^k^										
GAAGG	0.335	1.00	1.00	1.00	1.00	1.00	1.00	1.00	1.00	1.00
AGGGG	0.289	1.05 (0.74–1.48)	0.83 (0.54–1.29)	1.79 (0.98–3.28)	1.12 (0.81–1.56)	0.99 (0.65–1.52)	0.91 (0.49–1.70)	1.01 (0.61–1.66)	0.60 (0.30–1.20)	1.88 (0.80–4.45)
GAGGG	0.188	0.74 (0.51–1.07)	0.77 (0.49–1.21)	0.81 (0.40–1.63)	0.85 (0.59–1.21)	0.74 (0.46–1.19)	1.03 (0.55–1.94)	0.57 (0.32–1.02)	0.46 (0.22–0.93)	0.92 (0.30–2.79)
AGGTA	0.166	1.14 (0.77–1.68)	0.95 (0.58–1.56)	1.55 (0.79–3.03)	1.26 (0.89–1.80)	1.30 (0.83–2.05)	0.88 (0.47–1.67)	1.49 (0.88–2.51)	1.05 (0.53–2.08)	2.23 (0.87–5.68)
AGGTG	0.099	0.73 (0.45–1.19)	0.56 (0.29–1.08)	1.45 (0.69–3.03)	0.96 (0.62–1.50)	0.75 (0.41–1.39)	1.51 (0.71–3.18)	0.81 (0.41–1.59)	0.47 (1.14–1.58)	1.37 (0.57–3.32)
Global *P*^d^		0.283	0.584	0.473	0.575	0.583	0.931	0.219	0.300	0.728
*LEPR*, **block 9**^l^										
CG	0.653	1.00	1.00	1.00	1.00	1.00	1.00	1.00	1.00	1.00
TA	0.345	0.95 (0.72–1.26)	1.06 (0.76–1.49)	0.88 (0.52–1.50)	1.03 (0.79–1.33)	1.05 (0.76–1.46)	0.93 (0.58–1.52)	1.05 (0.70–1.58)	0.99 (0.58–1.68)	1.09 (0.53–2.24)
Global *P*^d^		0.954	0.918	0.975	0.996	0.979	0.994	0.997	1.000	0.997
*LEP*, **block 1**^m^										
TC	0.483	1.00	1.00	1.00	1.00	1.00	1.00	1.00	1.00	1.00
TT	0.448	1.23 (0.95–1.60)	1.12 (0.80–1.57)	1.46 (0.94–2.26)	1.38 (1.07–1.77)	1.53 (1.10–2.12)	0.98 (0.63–1.52)	1.33 (0.91–1.96)	1.60 (0.93–2.73)	1.14 (0.63–2.07)
CT	0.069	1.10 (0.68–1.79)	0.94 (0.53–1.67)	1.58 (0.60–4.17)	1.16 (0.73–1.84)	1.05 (0.60–1.84)	1.85 (0.74–4.64)	0.91 (0.38–2.19)	1.10 (0.40–3.00)	0.47 (0.06–3.77)
Global *P*^d^		0.483	0.884	0.377	0.097	0.072	0.623	0.470	0.382	0.807

CRC, colorectal cancer; HR, hazard ratio. Those with significant *P*-values after Bonferroni correction for 36 haplotypes are shown in bold (i.e., the adjusted *P*-value at the 0.05 significance level was 0.0014). ^a^Cox proportional hazard model adjusted for age at diagnosis, gender, race, stage at diagnosis, household income, reported screening procedure, marital status, family history, smoking status, alcohol consumption status, folate intake, MSI status, and BRAF mutation status, where applicable. ^b^Rare haplotypes with frequencies less than 1% were excluded from analyses. ^c^*LEPR*, block 1 includes rs12145690, rs4655802, and rs9436297. ^d^Global *P* for association is from a likelihood ratio test with degrees of freedom equal to the number of haplotypes. ^e^*LEPR*, block 2 includes rs7534511, rs9436301, and rs1887285. ^f^*LEPR*, block 3 includes rs7602, rs970467, and rs9436748. ^g^*LEPR*, block 4 includes rs6588147, rs7513047, rs17127673, and rs1327115. ^h^*LEPR*, block 5 includes rs11208659 and rs1475397. ^i^*LEPR*, block 6 includes rs1171278, rs1751492, rs6697315, and rs10749753. ^j^*LEPR*, block 7 includes rs10889562, rs1137100, rs10493380, and rs6673591. ^k^*LEPR*, block 8 includes rs10749754, rs1137101, rs4655537, rs12405556, and rs1938496. ^l^*LEPR*, block 9 includes rs1805096, and rs1892534. ^m^*LEP*, block 1 includes rs4731427, and rs11763517.

### Gene-environment interactions

To evaluate potential gene-environment interactions, we cross-tabulated red meat consumption, BMI, and *LEP* and *LEPR* polymorphisms among the participants (**Table 4**). Superior DFS was associated with *LEPR* rs7602 (A allele *vs*. G allele: HR, 0.48; 95% CI, 0.28–0.81) in the stratum of patients with red meat intake below the median. Additionally, *LEPR* rs1171278 (T allele *vs*. C allele: HR, 0.24; 95% CI, 0.09–0.62) was associated with greater prognostic benefits in patients with a BMI below than above 25 kg/m^2^.

**Table 4:** Associations between selected genetic variations in *LEP* or *LEPR* and colorectal cancer disease-free survival, stratified by red meat intake and BMI

Variant	Alleles^a^	Red meat HR (95% CI)^b^		*P*_int_^c^	BMI HR (95% CI)^b^		*P*_int_^c^
		< Median	≥ Median		< 25 kg/m^2^	≥ 25 kg/m^2^	
***LEPR***							
rs9436297	T/C	0.99 (0.63–1.55)	1.08 (0.66–1.77)	0.458	0.92 (0.42–1.99)	0.97 (0.67–1.41)	0.414
rs9436301	T/C	0.65 (0.42–1.01)	0.60 (0.39–0.91)	0.083	0.62 (0.36–1.05)	0.62 (0.44–0.88)	0.720
rs7602	G/A	0.48 (0.28–0.81)	0.64 (0.41–1.02)	0.020	0.52 (0.29–0.96)	0.60 (0.41–0.89)	0.950
rs17127673	A/G	0.52 (0.26–1.02)	0.71 (0.40–1.26)	0.105	0.29 (0.10–0.82)	0.83 (0.53–1.29)	0.066
rs4655537	G/A	1.00 (0.72–1.39)	1.06 (0.74–1.52)	0.628	0.90 (0.57–1.44)	1.02 (0.77–1.34)	0.333
rs1171278	C/T	0.54 (0.31–0.92)	0.61 (0.36–1.03)	0.086	0.24 (0.09–0.62)	0.84 (0.57–1.25)	0.025
rs1137100	A/G	1.00 (0.71–1.42)	1.12 (0.75–1.68)	0.482	0.95 (0.54–1.66)	1.04 (0.76–1.41)	0.625
rs1938496	G/A	0.94 (0.62–1.44)	0.63 (0.40–1.02)	0.276	0.77 (0.42–1.41)	0.81 (0.56–1.17)	0.405
***LEP***							
rs11763517	T/C	0.68 (0.48–0.96)	0.69 (0.46–1.02)	0.358	0.79 (0.49–1.28)	0.63 (0.46–0.86)	0.296

^a^Two variants at the locus are presented as major allele/minor allele. Hazard ratios were calculated with reference to the underlined allele. ^b^Cox proportional hazard model adjusted for age at diagnosis, gender, race, stage at diagnosis, household income, reported screening procedure, marital status, family history, smoking status, alcohol consumption status, folate intake, MSI status, and BRAF mutation status, where applicable. ^c^*P* for interaction is computed with the Wald method, testing the significance of multiplicative interaction terms between genetic variants and the respective stratified variable; not adjusted for multiple comparisons.

## Discussion

In this study, the *LEP* and *LEPR* genes were associated with DFS and CRC-specific survival in CRC, respectively, at the gene level. Notably, *LEP* rs11763517, *LEPR* rs9436301, and *LEPR* rs7602 polymorphisms exhibited significant associations with DFS in patients with CRC after adjustment for multiple comparisons. Haplotype analyses indicated that the *LEPR* block 2 haplotype G-C-T, defined by rs7534511, rs9436301, and rs1887285, and the block 3 haplotype A-A-G, defined by rs7602, rs970467, and rs9436748, were associated with prolonged OS and DFS among patients with CRC and colon cancer. Furthermore, the *LEP*/*LEPR*-CRC survival association appeared to be modified by red meat intake and BMI.

LEP, which acts as a growth factor in colonic epithelial cells, might underlie the observed associations among obesity, physical activity, and colon cancer^22^. Various polymorphisms in the *LEP* gene are associated with extreme obesity (BMI ≥ 40 kg/m^2^)^23^, and LEP concentration is positively correlated with BMI^24^. Leptin is an important adipokine believed to play critical roles in stimulating proliferation and inhibit apoptosis^25^. Previous studies have shown that LEP up-regulates miR-4443, thereby suppressing NCOA1 and TRAF4, and decreasing the invasiveness of human colon cancer cells^26^. Chronic increases in LEP concentration may enhance the growth of colonic cancers *via* the MAPK and PI3-K pathways^27^. *LEP* has been implicated in breast cancer, prostate cancer, and diffuse large B-cell lymphoma^28–30^. *LEP* and *LEPR* gene polymorphisms are associated with the risk of CRC^31,32^, but no studies have described *LEP* or *LEPR* polymorphisms and CRC survival. In the current study, we identified SNPs in the human *LEP* and *LEPR* genes associated with CRC survival, which have not been reported in previous studies. Specifically, rs11763517, which is located in the intron of *LEP* (hg19: chr7:127890062), was associated with prolonged DFS. Potential mechanisms underlying this observation may be that LEP influences the growth and survival of CRC stem cells, and regulates the adhesion and invasion of colorectal carcinoma cells through activation of the JAK and ERK signaling pathways^33^. However, the association between the *LEP* rs11763517 genotype and LEP expression remains to be determined. In-depth understanding of the mechanism between *LEP* rs11763517 and CRC survival will require further investigation.

The physiological mechanisms of LEP action are exerted through LEPR, which is often expressed in CRC. A cohort study has indicated that elevated LEPR expression is associated with increased neoangiogenesis and metastatic potential in CRC^34^; and the absence of *LEPR* expression is correlated with low rates of proliferation^35^. The rs7602 variant is in the intron of *LEPR* (hg19: chr1:65897951) and the 3′UTR of LEPR overlapping transcript (*LEPROT*); the protein encoded by *LEPROT* has been shown to negatively regulate LEPR expression in mice^36^. Kim et al.^37^ have demonstrated that *LEPR* rs7602 is significantly associated with the risk of late menarche associated with decreased CRC risk and all-cause mortality^38–40^. However, results from the NIH-AARP Diet and Health Study have not indicated the same association between reproductive/hormonal factors and CRC^41^. *LEPR* rs9436301 is a variant in the second intron of *LEPR* (hg19: chr1:65895927) that has been reported to be independently associated with *LEPR* expression levels^42^. Studies have shown that SNPs within introns have the potential to affect the alternative splicing of RNA^43^, and 3′UTR variants are strongly associated with human traits and diseases^44^. In the current study, none of the high-interest SNPs in the literature (rs1137101, rs1137100) were significantly associated with CRC survival. Haplotype analysis indicated that the haplotype G-C-T in block 2 and haplotype A-A-G in block 3 on *LEPR* were associated with prolonged OS and DFS among patients with CRC overall and patients with colon cancer, as compared with the most common haplotype. These 2 haplotypes contained SNPs that were significantly associated with CRC survival in the single SNP analysis. Notably, haplotype analysis provides insights into genetic diversity and thus may be superior to individual SNP analysis.

Furthermore, our study is the first to demonstrate that red meat intake and BMI status may modulate the relationships between *LEPR* rs7602 or *LEPR* rs1171278 and CRC survival. Although the mechanisms underlying the observed interactions are not yet fully understood, the positive associations among red meat intake, BMI, and LEP levels, as demonstrated in previous research^45,46^, may provide an explanation. In addition, we surmised that the influence of subtle differences among genotypes was overwhelmed by the detrimental effects of overweight/obesity or high red meat consumption on cancer outcomes. If the gene–environment interaction is replicated in future research, then CRC survivors, particularly those with high-risk genotypes, may benefit from behavioral interventions such as limiting red meat consumption while maintaining a healthful weight to improve their prognosis.

The strengths of this study include its moderately large sample size, the long period of follow-up, and the availability of detailed information on participants’ personal history. Our study also has several limitations. The information on diet and lifestyle habits was self-reported by participants when we started this investigation, thus potentially introducing bias due to misclassification; however, such bias would not have influenced the genetic findings. In addition, colorectal carcinogenesis is a long process during which disease may promote weight loss and patients may modify their food habits. Therefore, retrospective BMI and diet data might not reflect the real-life situation before death in patients with CRC. Future research assessing diet or dietary alterations post-diagnosis is needed to further elucidate possible gene-environment interactions in CRC outcomes.

## Conclusions

Overall, our study provides evidence that rs11763517 of the *LEP* gene, and rs9436301 and rs7602 of the *LEPR* gene are likely to be associated with CRC survival. The *LEP*/*LEPR*-CRC survival association was modified by red meat consumption and BMI. Notably, the SNPs examined in this study were tag SNPs, which are considered to be only indicators for specific regions of interest and thus may not reflect causality^47^. If our findings are successfully replicated in other well-powered studies, certain variants of the *LEP* and *LEPR* genes may serve as novel prognostic biomarkers for CRC, and CRC survivors may improve their prognosis through lifestyle changes.
