# Supplementary material for: Associations between polymorphisms in leptin and leptin receptor genes and colorectal cancer survival
Source: Cancer Biol Med. 2023 Jun 6;20(6):438–51. doi: 10.20892/j.issn.2095-3941.2022.0635 (PMC10291983; doi:10.20892/j.issn.2095-3941.2022.0635)
Supplement: Supplementary file 1 [file cbm-20-438-s001.pdf]

Supplementary materials

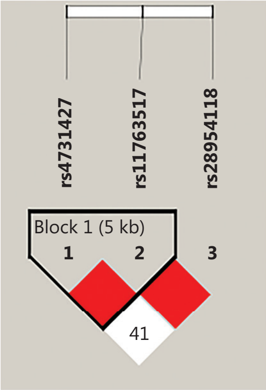

Figure S1 Linkage disequilibrium (LD) plot of *LEP* genes. LD strength between the SNPs is indicated with the standard Haploview color scheme based on both D´ and LOD values (D´ < 1 and LOD < 2 in white; D´ = 1 and LOD < 2 in blue; D´ < 1 and LOD ≥ 2 in shades of pink/red; D´ = 1 and LOD ≥ 2 in bright red). Numbers in squares are D´ (× 100), but those with D´ = 1 are not shown. The black triangle marks the single haplotype block within each gene.

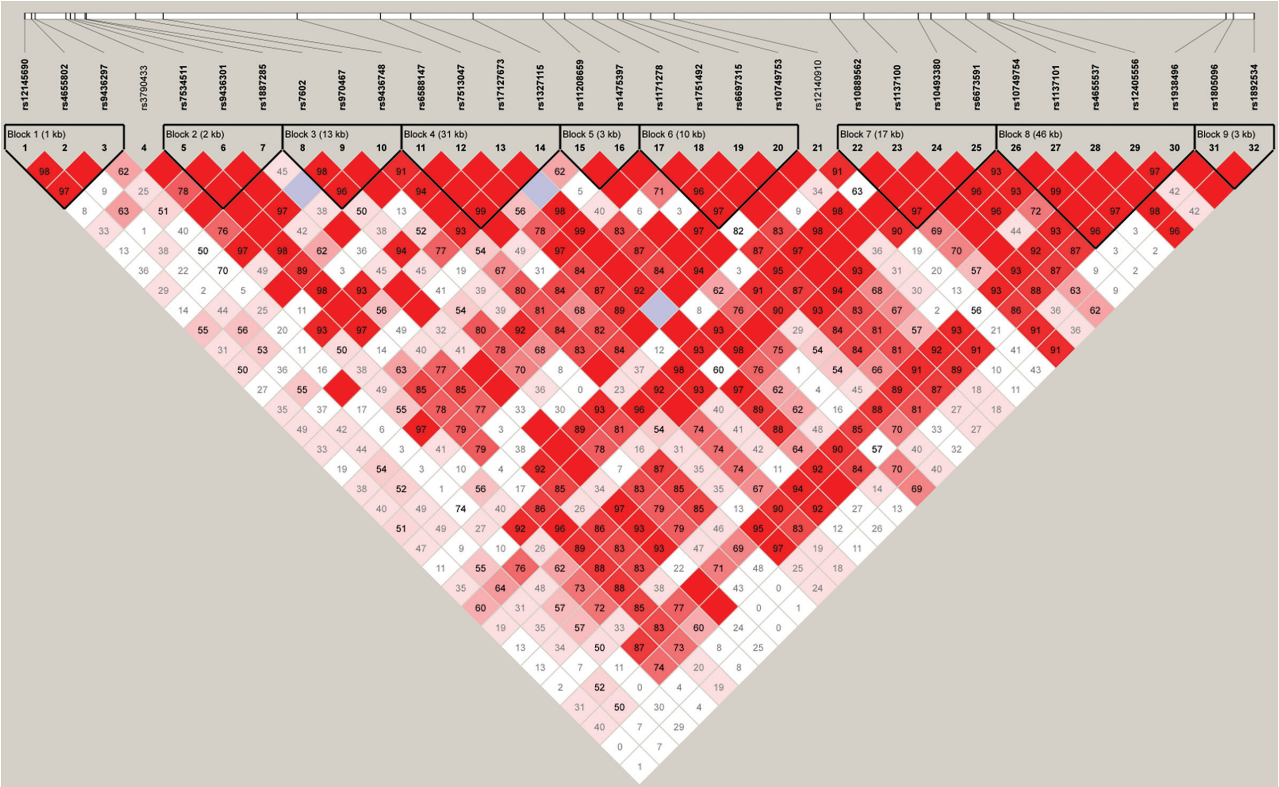

Figure S2 Linkage disequilibrium (LD) plot of *LEPR* genes. LD strength between SNPs is indicated with the standard Haploview color scheme based on both D´ and LOD values (D´ < 1 and LOD < 2 in white; D´ = 1 and LOD < 2 in blue; D′< 1 and LOD ≥ 2 in shades of pink/red; D´ = 1 and LOD ≥ 2 in bright red). Numbers in squares are D´ (× 100), but those with D´ = 1 are not shown. The black triangle marks the single haplotype block within each gene.

Table S1 Associations between *LEP* SNPs and colorectal cancer overall survival, disease-free survival, and CRC-specific survival, assuming an additive mode of inheritance

SNP ID	Position[a]	Major/minor allele (MAF)	All CRC			Colon cancer			Rectal cancer		
			HR (95% CI)[b]	*P*-value	P_{ACT}[d]	HR (95% CI)[b]	*P*-value	P_{ACT}[d]	HR (95% CI)[b]	*P*-value	P_{ACT}[d]
**Overall survival**[c]											
rs4731427	127884389	T/C (0.069)	1.02 (0.63–1.66)	0.934	1.000	1.14 (0.64–2.02)	0.663	1.000	0.79 (0.29–2.12)	0.636	1.000
rs11763517	127890062	T/C (0.483)	0.83 (0.65–1.08)	0.164	0.931	0.86 (0.63–1.20)	0.379	0.998	0.82 (0.53–1.26)	0.368	0.999
rs28954118	127895182	A/T (0.051)	1.04 (0.55–1.98)	0.897	1.000	1.07 (0.50–2.30)	0.863	1.000	1.32 (0.35–4.91)	0.683	1.000
**Disease-free survival**[c]											
rs4731427	127884389	T/C (0.069)	0.96 (0.61–1.50)	0.858	1.000	1.20 (0.69–2.08)	0.518	1.000	0.49 (0.21–1.13)	0.094	0.769
rs11763517	127890062	T/C (0.483)	0.64 (0.50–0.83)	0.001	0.015	0.63 (0.45–0.87)	0.006	0.118	0.67 (0.43–1.03)	0.069	0.684
rs28954118	127895182	A/T (0.051)	1.27 (0.69–2.32)	0.440	0.997	1.49 (0.69–3.19)	0.308	0.987	0.77 (0.26–2.25)	0.628	1.000
**CRC-specific survival**[c]											
rs4731427	127884389	T/C (0.065)	1.59 (0.65–3.93)	0.311	0.972	1.42 (0.50–4.02)	0.506	1.000	1.85 (0.22–15.91)	0.576	1.000
rs11763517	127890062	T/C (0.482)	0.76 (0.52–1.12)	0.166	0.870	0.59 (0.34–1.03)	0.070	0.660	0.97 (0.54–1.72)	0.906	1.000
rs28954118	127895182	A/T (0.051)	1.15 (0.41–3.23)	0.797	1.000	1.07 (0.31–3.67)	0.915	1.000	1.76 (0.17–18.19)	0.637	1.000

CRC, colorectal cancer; HR, hazard ratio; MAF, minor allele frequency. [a]SNP locations were mapped according to the NCBI build 36 coordinates. [b]Hazard ratios were calculated with reference to the underlined allele. [c]Cox proportional hazard model adjusted for age at diagnosis, gender, race, stage at diagnosis, household income, reported screening procedure, marital status, family history, smoking status, alcohol consumption status, folate intake, MSI status, and BRAF mutation status, where applicable. [d]*P*-values were adjusted for multiple comparisons with a modification of P_{ACT} for correlated tests developed by Conneely and Boehnke[21].

Table S2 Associations between *LEPR* SNPs and colorectal cancer overall survival, disease-free survival, and CRC-specific survival, assuming an additive mode of inheritance

SNP ID	Position[a]	Major/Minor allele (MAF)	All CRC			Colon cancer			Rectal cancer		
			HR (95% CI)[b]	*P*-value	P_{ACT}[d]	HR (95% CI)[b]	*P*-value	P_{ACT}[d]	HR (95% CI)[b]	*P*-value	P_{ACT}[d]
**Overall survival**[c]											
rs12145690	65887013	A/C (0.444)	0.96 (0.74–1.24)	0.731	1.000	0.93 (0.67–1.30)	0.685	1.000	0.98 (0.64–1.51)	0.925	1.000
rs4655802	65888231	A/G (0.428)	1.10 (0.86–1.41)	0.461	1.000	1.15 (0.84–1.58)	0.379	0.998	1.13 (0.72–1.78)	0.596	1.000
rs9436297	65888854	T/C (0.132)	1.13 (0.79–1.63)	0.498	1.000	1.21 (0.76–1.95)	0.422	0.997	1.20 (0.67–2.15)	0.551	1.000
rs3790433	65894342	G/A (0.267)	1.08 (0.83–1.42)	0.560	1.000	1.04 (0.75–1.43)	0.835	1.000	1.23 (0.70–2.14)	0.475	0.999
rs7534511	65895130	G/A (0.311)	0.99 (0.77–1.26)	0.906	1.000	1.10 (0.79–1.54)	0.562	1.000	0.86 (0.58–1.29)	0.467	1.000
rs9436301	65895927	T/C (0.271)	0.79 (0.59–1.05)	0.105	0.827	0.80 (0.56–1.13)	0.195	0.956	0.68 (0.38–1.21)	0.184	0.961
rs1887285	65897747	T/C (0.090)	0.95 (0.65–1.39)	0.778	1.000	0.84 (0.55–1.28)	0.415	0.998	1.57 (0.58–4.26)	0.375	0.999
rs7602	65897951	G/A (0.219)	0.71 (0.51–0.99)	0.044	0.557	0.69 (0.47–1.02)	0.060	0.652	0.74 (0.37–1.47)	0.384	0.998
rs970467	65906762	G/A (0.136)	1.50 (1.01–2.24)	0.045	0.558	1.56 (0.98–2.50)	0.063	0.665	1.41 (0.64–3.12)	0.401	0.999
rs9436748	65911672	G/T (0.426)	0.88 (0.69–1.13)	0.318	0.992	0.79 (0.58–1.08)	0.142	0.901	1.03 (0.67–1.57)	0.907	1.000
rs6588147	65935494	A/G (0.338)	0.96 (0.75–1.24)	0.777	1.000	0.84 (0.60–1.18)	0.308	0.994	1.11 (0.75–1.63)	0.599	1.000
rs7513047	65950340	G/A (0.476)	0.95 (0.74–1.21)	0.669	1.000	0.87 (0.64–1.19)	0.382	0.997	1.05 (0.69–1.60)	0.823	1.000

Table S2 Continued

SNP ID	Position[a]	Major/Minor allele (MAF)	All CRC			Colon cancer			Rectal cancer		
			HR (95% CI)[b]	*P*-value	P_{ACT}[d]	HR (95% CI)[b]	*P*-value	P_{ACT}[d]	HR (95% CI)[b]	*P*-value	P_{ACT}[d]
rs17127673	65955725	A/G (0.115)	0.75 (0.49–1.13)	0.171	0.927	0.78 (0.47–1.28)	0.324	0.995	0.67 (0.31–1.45)	0.305	0.996
rs1327115	65966839	G/T (0.410)	1.05 (0.83–1.33)	0.663	1.000	0.97 (0.71–1.32)	0.835	1.000	1.16 (0.80–1.68)	0.446	0.999
rs11208659	65979280	T/C (0.095)	0.87 (0.59–1.28)	0.480	1.000	0.81 (0.50–1.31)	0.387	0.997	0.81 (0.40–1.67)	0.573	1.000
rs1475397	65983158	C/T (0.254)	0.92 (0.69–1.22)	0.549	1.000	0.93 (0.66–1.31)	0.674	1.000	0.96 (0.57–1.61)	0.865	1.000
rs1171278	65988146	C/T (0.160)	0.77 (0.53–1.11)	0.163	0.932	0.75 (0.48–1.18)	0.210	0.964	0.78 (0.40–1.51)	0.459	1.000
rs1751492	65992625	T/C (0.294)	1.09 (0.83–1.44)	0.518	1.000	1.16 (0.81–1.67)	0.416	0.997	1.00 (0.66–1.52)	0.999	1.000
rs6697315	65993556	T/C (0.368)	0.95 (0.74–1.21)	0.668	1.000	0.96 (0.70–1.32)	0.806	1.000	0.91 (0.61–1.36)	0.636	1.000
rs10749753	65998467	A/G (0.481)	1.08 (0.84–1.39)	0.544	1.000	1.11 (0.82–1.52)	0.492	0.999	1.02 (0.66–1.59)	0.917	1.000
rs12140910	66002660	A/G (0.074)	1.49 (1.00–2.23)	0.052	0.600	1.54 (0.94–2.50)	0.084	0.754	1.56 (0.70–3.48)	0.276	0.994
rs10889562	66030445	G/A (0.240)	0.86 (0.65–1.12)	0.262	0.981	0.89 (0.62–1.27)	0.516	1.000	0.84 (0.52–1.35)	0.470	1.000
rs1137100	66036441	A/G (0.265)	0.92 (0.69–1.23)	0.584	1.000	0.84 (0.58–1.23)	0.375	0.998	1.02 (0.65–1.59)	0.929	1.000
rs10493380	66046117	A/C (0.190)	0.90 (0.67–1.22)	0.512	1.000	0.99 (0.68–1.46)	0.975	1.000	0.89 (0.48–1.63)	0.698	1.000
rs6673591	66048389	G/A (0.480)	1.03 (0.81–1.30)	0.816	1.000	0.94 (0.70–1.27)	0.705	1.000	1.16 (0.77–1.76)	0.479	0.999
rs10749754	66054640	G/A (0.474)	1.02 (0.80–1.29)	0.878	1.000	1.26 (0.94–1.70)	0.130	0.879	0.67 (0.43–1.05)	0.079	0.759
rs1137101	66058513	A/G (0.471)	1.00 (0.79–1.26)	0.988	1.000	0.81 (0.60–1.09)	0.162	0.926	1.49 (0.96–2.31)	0.079	0.757
rs4655537	66058801	G/A (0.340)	0.85 (0.67–1.08)	0.184	0.936	0.75 (0.56–0.99)	0.045	0.563	1.24 (0.77–2.01)	0.375	0.999
rs12405556	66063117	G/T (0.265)	1.06 (0.80–1.41)	0.678	1.000	1.20 (0.82–1.76)	0.350	0.997	0.93 (0.60–1.44)	0.728	1.000
rs1938496	66100915	G/A (0.169)	0.90 (0.63–1.29)	0.559	1.000	1.00 (0.63–1.58)	0.987	1.000	0.84 (0.46–1.53)	0.564	1.000
rs1805096	66102257	C/T (0.345)	0.93 (0.72–1.21)	0.597	1.000	1.00 (0.72–1.37)	0.974	1.000	0.86 (0.52–1.43)	0.569	1.000
rs1892534	66105944	G/A (0.347)	0.93 (0.72–1.21)	0.597	1.000	0.99 (0.72–1.37)	0.967	1.000	0.86 (0.52–1.43)	0.569	1.000
**Disease-free survival**[c]											
rs12145690	65887013	A/C (0.444)	0.98 (0.78–1.24)	0.891	1.000	1.02 (0.75–1.37)	0.923	1.000	1.04 (0.71–1.53)	0.839	1.000
rs4655802	65888231	A/G (0.428)	1.06 (0.84–1.34)	0.624	1.000	1.00 (0.75–1.34)	0.995	1.000	1.09 (0.73–1.62)	0.670	1.000
rs9436297	65888854	T/C (0.132)	1.08 (0.78–1.50)	0.627	1.000	1.05 (0.70–1.60)	0.804	1.000	1.21 (0.71–2.04)	0.483	0.998
rs3790433	65894342	G/A (0.267)	1.30 (0.99–1.69)	0.058	0.590	1.21 (0.87–1.67)	0.255	0.974	1.40 (0.83–2.39)	0.211	0.946
rs7534511	65895130	G/A (0.311)	0.85 (0.67–1.08)	0.187	0.911	0.84 (0.62–1.15)	0.282	0.981	0.83 (0.56–1.25)	0.374	0.993
rs9436301	65895927	T/C (0.271)	0.60 (0.45–0.79)	0.000	0.010	0.62 (0.44–0.89)	0.009	0.159	0.56 (0.31–0.99)	0.048	0.570
rs1887285	65897747	T/C (0.090)	1.13 (0.76–1.66)	0.550	1.000	0.95 (0.61–1.49)	0.833	1.000	1.57 (0.65–3.76)	0.317	0.986
rs7602	65897951	G/A (0.219)	0.55 (0.39–0.76)	0.000	0.008	0.57 (0.38–0.85)	0.006	0.114	0.54 (0.28–1.05)	0.068	0.684
rs970467	65906762	G/A (0.136)	1.77 (1.19–2.62)	0.005	0.091	1.77 (1.11–2.83)	0.017	0.272	1.68 (0.77–3.66)	0.193	0.937
rs9436748	65911672	G/T (0.426)	0.89 (0.71–1.12)	0.330	0.985	0.94 (0.70–1.26)	0.669	1.000	0.91 (0.60–1.36)	0.630	1.000
rs6588147	65935494	A/G (0.338)	1.04 (0.82–1.32)	0.734	1.000	1.04 (0.76–1.42)	0.809	1.000	1.14 (0.77–1.67)	0.521	1.000
rs7513047	65950340	G/A (0.476)	0.92 (0.73–1.16)	0.489	0.998	0.98 (0.73–1.31)	0.878	1.000	0.89 (0.60–1.34)	0.584	1.000
rs17127673	65955725	A/G (0.115)	0.61 (0.40–0.91)	0.016	0.246	0.69 (0.42–1.12)	0.133	0.851	0.46 (0.22–0.97)	0.041	0.531

Table S2 Continued

SNP ID	Position[a]	Major/Minor allele (MAF)	All CRC			Colon cancer			Rectal cancer		
			HR (95% CI)[b]	*P*-value	P_{ACT}[d]	HR (95% CI)[b]	*P*-value	P_{ACT}[d]	HR (95% CI)[b]	*P*-value	P_{ACT}[d]
rs1327115	65966839	G/T (0.410)	1.11 (0.89–1.39)	0.373	0.992	1.14 (0.85–1.52)	0.386	0.994	1.15 (0.80–1.66)	0.446	0.997
rs11208659	65979280	T/C (0.095)	0.82 (0.57–1.19)	0.296	0.981	0.78 (0.48–1.26)	0.311	0.983	0.91 (0.48–1.70)	0.759	1.000
rs1475397	65983158	C/T (0.254)	0.81 (0.62–1.06)	0.124	0.804	0.86 (0.62–1.21)	0.398	0.994	0.73 (0.46–1.17)	0.190	0.938
rs1171278	65988146	C/T (0.160)	0.60 (0.42–0.86)	0.006	0.106	0.67 (0.43–1.03)	0.070	0.657	0.52 (0.28–0.99)	0.047	0.572
rs1751492	65992625	T/C (0.294)	0.92 (0.72–1.18)	0.519	1.000	0.91 (0.66–1.26)	0.574	1.000	0.90 (0.59–1.38)	0.626	1.000
rs6697315	65993556	T/C (0.368)	0.83 (0.65–1.04)	0.109	0.762	0.78 (0.58–1.06)	0.111	0.808	0.85 (0.56–1.27)	0.419	0.995
rs10749753	65998467	A/G (0.481)	1.08 (0.85–1.37)	0.528	1.000	1.01 (0.75–1.36)	0.956	1.000	1.15 (0.76–1.73)	0.503	1.000
rs12140910	66002660	A/G (0.074)	1.43 (0.98–2.10)	0.068	0.623	1.60 (0.99–2.58)	0.056	0.588	1.27 (0.62–2.58)	0.516	1.000
rs10889562	66030445	G/A (0.240)	0.80 (0.61–1.04)	0.090	0.713	0.77 (0.55–1.08)	0.129	0.851	0.83 (0.54–1.29)	0.414	0.996
rs1137100	66036441	A/G (0.265)	1.06 (0.82–1.38)	0.658	1.000	1.06 (0.76–1.48)	0.727	1.000	1.15 (0.74–1.80)	0.542	1.000
rs10493380	66046117	A/C (0.190)	0.95 (0.71–1.26)	0.705	1.000	0.88 (0.61–1.29)	0.519	1.000	1.15 (0.69–1.91)	0.588	1.000
rs6673591	66048389	G/A (0.480)	1.22 (0.97–1.53)	0.094	0.716	1.23 (0.92–1.65)	0.161	0.894	1.26 (0.84–1.91)	0.264	0.973
rs10749754	66054640	G/A (0.474)	0.86 (0.68–1.08)	0.197	0.920	0.95 (0.72–1.26)	0.720	1.000	0.65 (0.43–1.00)	0.052	0.598
rs1137101	66058513	A/G (0.471)	1.17 (0.93–1.48)	0.172	0.892	1.07 (0.80–1.41)	0.662	1.000	1.53 (1.00–2.36)	0.052	0.597
rs4655537	66058801	G/A (0.340)	1.02 (0.81–1.29)	0.853	1.000	0.94 (0.71–1.25)	0.673	1.000	1.29 (0.82–2.02)	0.276	0.977
rs12405556	66063117	G/T (0.265)	0.90 (0.70–1.17)	0.450	0.996	0.90 (0.65–1.26)	0.546	1.000	0.87 (0.56–1.35)	0.519	1.000
rs1938496	66100915	G/A (0.169)	0.74 (0.54–1.01)	0.060	0.594	0.66 (0.44–0.97)	0.033	0.426	0.99 (0.57–1.69)	0.956	1.000
rs1805096	66102257	C/T (0.345)	1.04 (0.82–1.32)	0.734	1.000	1.14 (0.85–1.53)	0.385	0.994	0.88 (0.56–1.36)	0.551	1.000
rs1892534	66105944	G/A (0.347)	1.04 (0.82–1.31)	0.749	1.000	1.13 (0.84–1.51)	0.428	0.996	0.88 (0.56–1.36)	0.551	1.000
**CRC-specific survival**[c]											
rs12145690	65887013	A/C (0.452)	1.05 (0.70–1.57)	0.813	1.000	1.10 (0.62–1.97)	0.744	1.000	1.09 (0.59–2.02)	0.788	1.000
rs4655802	65888231	A/G (0.425)	1.18 (0.79–1.77)	0.430	0.985	1.39 (0.79–2.42)	0.253	0.943	1.21 (0.61–2.42)	0.588	1.000
rs9436297	65888854	T/C (0.126)	1.55 (0.85–2.82)	0.153	0.879	2.81 (1.09–7.24)	0.033	0.461	1.55 (0.67–3.58)	0.309	0.988
rs3790433	65894342	G/A (0.264)	1.55 (1.01–2.37)	0.043	0.506	1.64 (0.98–2.77)	0.062	0.631	1.32 (0.59–2.97)	0.502	1.000
rs7534511	65895130	G/A (0.326)	0.69 (0.49–0.99)	0.042	0.505	0.60 (0.35–1.03)	0.064	0.636	0.76 (0.44–1.30)	0.313	0.986
rs9436301	65895927	T/C (0.270)	0.55 (0.35–0.87)	0.011	0.192	0.55 (0.31–0.96)	0.035	0.463	0.57 (0.23–1.39)	0.215	0.956
rs1887285	65897747	T/C (0.084)	1.70 (0.92–3.16)	0.092	0.739	1.53 (0.76–3.06)	0.230	0.939	2.05 (0.42–9.98)	0.373	0.991
rs7602	65897951	G/A (0.219)	0.42 (0.24–0.74)	0.003	0.062	0.41 (0.20–0.83)	0.013	0.227	0.39 (0.13–1.22)	0.106	0.831
rs970467	65906762	G/A (0.142)	1.58 (0.85–2.94)	0.153	0.864	1.57 (0.76–3.24)	0.219	0.940	2.48 (0.6–10.20)	0.210	0.957
rs9436748	65911672	G/T (0.413)	0.97 (0.67–1.39)	0.859	1.000	0.90 (0.54–1.50)	0.674	1.000	1.07 (0.61–1.89)	0.814	1.000
rs6588147	65935494	A/G (0.352)	1.17 (0.81–1.69)	0.404	0.986	1.24 (0.70–2.19)	0.464	0.997	1.12 (0.64–1.95)	0.703	1.000
rs7513047	65950340	G/A (0.462)	1.07 (0.73–1.57)	0.718	1.000	1.10 (0.66–1.84)	0.712	1.000	1.02 (0.55–1.88)	0.960	1.000
rs17127673	65955725	A/G (0.112)	0.39 (0.18–0.83)	0.014	0.240	0.43 (0.17–1.10)	0.078	0.676	0.25 (0.06–1.15)	0.076	0.734
rs1327115	65966839	G/T (0.426)	1.41 (0.99–2.01)	0.059	0.602	1.57 (0.91–2.71)	0.105	0.768	1.33 (0.77–2.27)	0.305	0.988

Table S2 Continued

SNP ID	Position[a]	Major/Minor allele (MAF)	All CRC			Colon cancer			Rectal cancer		
			HR (95% CI)[b]	*P*-value	P_{ACT}[d]	HR (95% CI)[b]	*P*-value	P_{ACT}[d]	HR (95% CI)[b]	*P*-value	P_{ACT}[d]
rs11208659	65979280	T/C (0.095)	0.60 (0.34–1.07)	0.083	0.720	0.60 (0.27–1.30)	0.193	0.923	0.47 (0.17–1.30)	0.145	0.890
rs1475397	65983158	C/T (0.250)	0.76 (0.47–1.22)	0.250	0.945	0.79 (0.43–1.45)	0.450	0.997	0.62 (0.26–1.47)	0.279	0.983
rs1171278	65988146	C/T (0.156)	0.39 (0.20–0.76)	0.005	0.108	0.41 (0.17–0.97)	0.042	0.514	0.18 (0.05–0.67)	0.011	0.205
rs1751492	65992625	T/C (0.306)	0.84 (0.57–1.25)	0.392	0.986	0.81 (0.45–1.48)	0.499	0.998	0.78 (0.42–1.45)	0.431	0.995
rs6697315	65993556	T/C (0.380)	0.70 (0.49–1.01)	0.053	0.568	0.64 (0.37–1.11)	0.114	0.793	0.64 (0.35–1.16)	0.139	0.881
rs10749753	65998467	A/G (0.474)	1.01 (0.68–1.50)	0.946	1.000	0.95 (0.56–1.62)	0.856	1.000	1.13 (0.59–2.16)	0.720	1.000
rs12140910	66002660	A/G (0.074)	1.90 (1.05–3.44)	0.033	0.437	1.85 (0.85–4.01)	0.121	0.803	2.31 (0.82–6.48)	0.113	0.837
rs10889562	66030445	G/A (0.234)	0.87 (0.58–1.31)	0.509	1.000	1.06 (0.56–2.00)	0.868	1.000	0.77 (0.43–1.37)	0.366	0.993
rs1137100	66036441	A/G (0.281)	1.23 (0.81–1.87)	0.336	0.971	1.21 (0.65–2.24)	0.545	1.000	1.40 (0.71–2.75)	0.329	0.988
rs10493380	66046117	A/C (0.192)	0.95 (0.58–1.54)	0.825	1.000	0.97 (0.52–1.81)	0.912	1.000	0.94 (0.38–2.32)	0.886	1.000
rs6673591	66048389	G/A (0.473)	1.26 (0.89–1.79)	0.192	0.889	1.06 (0.66–1.71)	0.801	1.000	1.72 (0.94–3.12)	0.077	0.730
rs10749754	66054640	G/A (0.484)	0.89 (0.62–1.28)	0.543	1.000	1.20 (0.74–1.94)	0.467	0.996	0.51 (0.27–0.99)	0.046	0.565
rs1137101	66058513	A/G (0.482)	1.14 (0.80–1.62)	0.485	0.991	0.85 (0.53–1.38)	0.520	1.000	1.95 (1.01–3.75)	0.046	0.564
rs4655537	66058801	G/A (0.330)	0.86 (0.61–1.23)	0.412	0.985	0.64 (0.42–1.00)	0.048	0.553	1.76 (0.88–3.54)	0.111	0.839
rs12405556	66063117	G/T (0.281)	0.78 (0.51–1.19)	0.252	0.940	0.80 (0.43–1.51)	0.492	0.998	0.68 (0.35–1.33)	0.260	0.977
rs1938496	66100915	G/A (0.180)	0.56 (0.34–0.93)	0.026	0.373	0.58 (0.30–1.15)	0.119	0.806	0.53 (0.22–1.23)	0.139	0.886
rs1805096	66102257	C/T (0.358)	1.10 (0.76–1.61)	0.607	1.000	1.10 (0.66–1.83)	0.708	1.000	1.26 (0.63–2.50)	0.509	1.000
rs1892534	66105944	G/A (0.360)	1.10 (0.76–1.61)	0.607	1.000	1.10 (0.66–1.83)	0.708	1.000	1.26 (0.63–2.50)	0.509	1.000

CRC, colorectal cancer; HR, hazard ratio; MAF, minor allele frequency. [a]SNP locations were mapped according to the NCBI build 36 coordinates. [b]Hazard ratios were calculated with reference to the underlined allele. [c]Cox proportional hazard model adjusted for age at diagnosis, gender, race, stage at diagnosis, household income, reported screening procedure, marital status, family history, smoking status, alcohol consumption status, folate intake, MSI status, and BRAF mutation status, where applicable. [d]*P*-values were adjusted for multiple comparisons with a modification of P_{ACT} for correlated tests developed by Conneely and Boehnke[21].

Table S3 Associations between *LEPR* SNPs and colorectal cancer overall survival, disease-free survival, and CRC-specific survival after stage at diagnosis stratification

SNP ID	Position[a]	Major/Minor allele (MAF)	All CRC			I/II			III/IV		
			HR (95% CI)[b]	*P*-value	P_{ACT}[d]	HR (95% CI)[b]	*P*-value	P_{ACT}[d]	HR (95% CI)[b]	*P*-value	P_{ACT}[d]
**Overall survival**[c]											
rs12145690	65887013	A/C (0.444)	0.94 (0.72–1.22)	0.630	1.000	0.90 (0.64–1.26)	0.523	1.000	1.01 (0.65–1.58)	0.953	0.960
rs4655802	65888231	A/G (0.428)	1.12 (0.87–1.44)	0.368	0.997	1.19 (0.87–1.64)	0.277	0.980	1.12 (0.71–1.77)	0.632	0.998
rs9436297	65888854	T/C (0.132)	1.13 (0.78–1.62)	0.526	1.000	1.22 (0.76–1.96)	0.417	0.998	1.17 (0.65–2.10)	0.606	0.997
rs3790433	65894342	G/A (0.267)	1.10 (0.84–1.45)	0.492	0.999	1.05 (0.76–1.45)	0.765	1.000	1.28 (0.72–2.25)	0.400	0.955
rs7534511	65895130	G/A (0.311)	1.00 (0.78–1.29)	0.994	1.000	1.17 (0.77–1.79)	0.470	0.999	0.88 (0.63–1.24)	0.473	0.977
rs9436301	65895927	T/C (0.271)	0.79 (0.59–1.05)	0.107	0.833	1.00 (0.61–1.62)	0.986	1.000	0.68 (0.46–1.01)	0.058	0.631
rs1887285	65897747	T/C (0.090)	0.93 (0.64–1.37)	0.726	1.000	0.52 (0.27–1.01)	0.053	0.614	1.06 (0.64–1.77)	0.816	1.000

Table S3 Continued

SNP ID	Position[a]	Major/Minor allele (MAF)	All CRC			I/II			III/IV		
			HR (95% CI)[b]	*P*-value	P_{ACT}^{d}	HR (95% CI)[b]	*P*-value	P_{ACT}^{d}	HR (95% CI)[b]	*P*-value	P_{ACT}^{d}
rs7602	65897951	G/A (0.219)	0.71 (0.51–0.99)	0.040	0.521	1.08 (0.67–1.74)	0.755	1.000	0.45 (0.28–0.74)	0.002	0.035
rs970467	65906762	G/A (0.136)	1.53 (1.02–2.30)	0.038	0.511	1.20 (0.64–2.23)	0.573	1.000	1.70 (0.94–3.10)	0.081	0.743
rs9436748	65911672	G/T (0.426)	0.86 (0.67–1.10)	0.218	0.960	0.89 (0.61–1.29)	0.538	1.000	0.78 (0.56–1.10)	0.158	0.892
rs6588147	65935494	A/G (0.338)	0.95 (0.74–1.22)	0.690	1.000	0.75 (0.49–1.15)	0.182	0.942	1.01 (0.71–1.42)	0.978	0.979
rs7513047	65950340	G/A (0.476)	0.93 (0.73–1.19)	0.562	1.000	1.00 (0.67–1.48)	0.988	1.000	0.84 (0.60–1.18)	0.310	0.948
rs17127673	65955725	A/G (0.115)	0.76 (0.50–1.16)	0.201	0.952	1.88 (1.06–3.37)	0.032	0.460	0.49 (0.26–0.93)	0.030	0.426
rs1327115	65966839	G/T (0.410)	1.03 (0.81–1.31)	0.816	1.000	0.77 (0.51–1.15)	0.205	0.956	1.08 (0.78–1.49)	0.650	1.000
rs11208659	65979280	T/C (0.095)	0.90 (0.61–1.33)	0.591	1.000	1.10 (0.54–2.26)	0.795	1.000	0.70 (0.42–1.14)	0.152	0.867
rs1475397	65983158	C/T (0.254)	0.91 (0.69–1.21)	0.520	1.000	1.35 (0.86–2.12)	0.200	0.956	0.70 (0.46–1.07)	0.095	0.783
rs1171278	65988146	C/T (0.160)	0.78 (0.54–1.13)	0.191	0.946	1.56 (0.93–2.61)	0.092	0.769	0.40 (0.22–0.70)	0.002	0.037
rs1751492	65992625	T/C (0.294)	1.12 (0.85–1.47)	0.429	0.998	1.50 (0.92–2.45)	0.102	0.796	0.74 (0.51–1.08)	0.115	0.835
rs6697315	65993556	T/C (0.368)	0.98 (0.76–1.25)	0.846	1.000	1.28 (0.83–1.97)	0.269	0.978	0.72 (0.51–1.00)	0.052	0.600
rs10749753	65998467	A/G (0.481)	1.11 (0.86–1.42)	0.430	0.998	1.01 (0.68–1.52)	0.955	1.000	1.04 (0.73–1.49)	0.837	1.000
rs12140910	66002660	A/G (0.074)	1.45 (0.96–2.18)	0.078	0.740	1.37 (0.67–2.77)	0.387	0.997	1.29 (0.75–2.23)	0.364	0.957
rs10889562	66030445	G/A (0.240)	0.85 (0.65–1.12)	0.253	0.977	0.96 (0.62–1.47)	0.833	1.000	0.94 (0.63–1.41)	0.772	1.000
rs1137100	66036441	A/G (0.265)	0.90 (0.68–1.21)	0.486	0.999	0.75 (0.46–1.21)	0.233	0.970	1.12 (0.76–1.65)	0.577	0.993
rs10493380	66046117	A/C (0.190)	0.90 (0.67–1.22)	0.510	1.000	0.88 (0.53–1.46)	0.623	1.000	0.93 (0.60–1.44)	0.739	1.000
rs6673591	66048389	G/A (0.480)	1.01 (0.80–1.29)	0.911	1.000	0.81 (0.55–1.19)	0.285	0.981	1.23 (0.88–1.72)	0.224	0.932
rs10749754	66054640	G/A (0.474)	1.05 (0.83–1.34)	0.684	1.000	1.14 (0.77–1.70)	0.520	1.000	0.97 (0.68–1.37)	0.847	1.000
rs1137101	66058513	A/G (0.471)	0.97 (0.76–1.23)	0.792	1.000	0.90 (0.61–1.34)	0.608	1.000	1.03 (0.73–1.47)	0.850	1.000
rs4655537	66058801	G/A (0.340)	0.84 (0.66–1.06)	0.145	0.909	0.79 (0.54–1.15)	0.220	0.965	0.88 (0.62–1.26)	0.488	0.990
rs12405556	66063117	G/T (0.265)	1.11 (0.83–1.48)	0.484	0.999	1.21 (0.76–1.92)	0.427	0.997	0.97 (0.65–1.45)	0.877	1.000
rs1938496	66100915	G/A (0.169)	0.97 (0.67–1.41)	0.882	1.000	0.88 (0.51–1.50)	0.635	1.000	1.09 (0.65–1.82)	0.754	1.000
rs1805096	66102257	C/T (0.345)	0.91 (0.70–1.19)	0.494	0.999	1.08 (0.70–1.67)	0.724	1.000	0.78 (0.54–1.13)	0.190	0.894
rs1892534	66105944	G/A (0.347)	0.91 (0.70–1.19)	0.495	0.999	1.08 (0.70–1.67)	0.724	1.000	0.80 (0.56–1.16)	0.238	0.957
**Disease-free survival**[c]											
rs12145690	65887013	A/C (0.444)	0.85 (0.66–1.10)	0.218	0.961	1.08 (0.76–1.52)	0.218	0.960	0.88 (0.64–1.22)	0.453	0.993
rs4655802	65888231	A/G (0.428)	1.11 (0.86–1.44)	0.424	0.998	0.99 (0.69–1.43)	0.424	0.996	1.01 (0.73–1.40)	0.940	1.000
rs9436297	65888854	T/C (0.132)	0.91 (0.63–1.31)	0.611	1.000	1.10 (0.61–1.98)	0.611	1.000	0.88 (0.58–1.34)	0.551	1.000
rs3790433	65894342	G/A (0.267)	1.01 (0.76–1.35)	0.953	1.000	1.04 (0.66–1.66)	0.953	1.000	1.27 (0.89–1.80)	0.190	0.910
rs7534511	65895130	G/A (0.311)	1.02 (0.79–1.32)	0.893	1.000	0.75 (0.51–1.10)	0.893	1.000	0.79 (0.56–1.12)	0.181	0.901
rs9436301	65895927	T/C (0.271)	0.83 (0.61–1.13)	0.234	0.962	0.78 (0.49–1.24)	0.234	0.962	0.55 (0.37–0.82)	0.003	0.065
rs1887285	65897747	T/C (0.090)	0.74 (0.50–1.12)	0.152	0.905	0.67 (0.34–1.34)	0.152	0.919	1.16 (0.68–1.97)	0.585	1.000
rs7602	65897951	G/A (0.219)	0.72 (0.51–1.00)	0.052	0.597	0.82 (0.51–1.31)	0.052	0.617	0.39 (0.24–0.63)	0.000	0.003

Table S3 Continued

SNP ID	Position[a]	Major/Minor allele (MAF)	All CRC			I/II			III/IV		
			HR (95% CI)[b]	*P*-value	P_{ACT}^{d}	HR (95% CI)[b]	*P*-value	P_{ACT}^{d}	HR (95% CI)[b]	*P*-value	P_{ACT}^{d}
rs970467	65906762	G/A (0.136)	1.45 (0.94–2.22)	0.090	0.777	1.51 (0.78–2.92)	0.090	0.796	1.86 (1.07–3.25)	0.028	0.381
rs9436748	65911672	G/T (0.426)	0.82 (0.65–1.05)	0.119	0.846	1.06 (0.74–1.52)	0.119	0.863	0.79 (0.57–1.10)	0.151	0.858
rs6588147	65935494	A/G (0.338)	0.86 (0.66–1.13)	0.276	0.979	1.12 (0.75–1.68)	0.276	0.979	0.94 (0.67–1.33)	0.741	1.000
rs7513047	65950340	G/A (0.476)	0.92 (0.72–1.18)	0.518	1.000	1.06 (0.73–1.55)	0.518	1.000	0.82 (0.59–1.14)	0.247	0.954
rs17127673	65955725	A/G (0.115)	0.95 (0.63–1.44)	0.813	1.000	0.95 (0.50–1.80)	0.813	1.000	0.51 (0.28–0.91)	0.022	0.238
rs1327115	65966839	G/T (0.410)	0.94 (0.73–1.20)	0.621	1.000	1.09 (0.74–1.59)	0.621	1.000	1.08 (0.78–1.50)	0.628	1.000
rs11208659	65979280	T/C (0.095)	0.78 (0.52–1.16)	0.222	0.960	1.22 (0.63–2.36)	0.222	0.960	0.62 (0.38–0.98)	0.043	0.487
rs1475397	65983158	C/T (0.254)	1.02 (0.76–1.38)	0.884	1.000	0.81 (0.52–1.26)	0.884	1.000	0.74 (0.50–1.08)	0.115	0.801
rs1171278	65988146	C/T (0.160)	0.85 (0.59–1.23)	0.381	0.995	0.83 (0.49–1.40)	0.381	0.995	0.40 (0.24–0.68)	0.001	0.015
rs1751492	65992625	T/C (0.294)	1.08 (0.82–1.43)	0.590	1.000	0.93 (0.60–1.44)	0.590	1.000	0.82 (0.58–1.16)	0.262	0.960
rs6697315	65993556	T/C (0.368)	0.95 (0.74–1.23)	0.697	1.000	0.89 (0.60–1.33)	0.697	1.000	0.74 (0.53–1.01)	0.061	0.596
rs10749753	65998467	A/G (0.481)	1.02 (0.79–1.32)	0.879	1.000	1.00 (0.68–1.46)	0.879	1.000	1.11 (0.80–1.55)	0.529	0.992
rs12140910	66002660	A/G (0.074)	1.43 (0.94–2.16)	0.097	0.790	1.15 (0.59–2.23)	0.097	0.809	1.50 (0.88–2.54)	0.136	0.844
rs10889562	66030445	G/A (0.240)	0.98 (0.74–1.30)	0.866	1.000	0.98 (0.65–1.48)	0.866	1.000	0.71 (0.49–1.03)	0.072	0.653
rs1137100	66036441	A/G (0.265)	0.88 (0.66–1.18)	0.389	0.995	1.27 (0.81–2.00)	0.389	0.992	1.05 (0.73–1.52)	0.787	1.000
rs10493380	66046117	A/C (0.190)	0.96 (0.70–1.32)	0.796	1.000	0.97 (0.60–1.57)	0.796	1.000	0.90 (0.60–1.35)	0.607	1.000
rs6673591	66048389	G/A (0.480)	0.96 (0.75–1.23)	0.744	1.000	1.13 (0.77–1.65)	0.744	1.000	1.42 (1.02–1.96)	0.037	0.448
rs10749754	66054640	G/A (0.474)	1.13 (0.87–1.45)	0.359	0.995	0.77 (0.52–1.14)	0.359	0.995	0.83 (0.60–1.15)	0.265	0.960
rs1137101	66058513	A/G (0.471)	0.91 (0.70–1.16)	0.436	0.997	1.32 (0.90–1.95)	0.436	0.995	1.21 (0.87–1.69)	0.257	0.958
rs4655537	66058801	G/A (0.340)	0.84 (0.65–1.07)	0.164	0.913	1.13 (0.77–1.65)	0.164	0.913	1.07 (0.75–1.51)	0.721	1.000
rs12405556	66063117	G/T (0.265)	1.10 (0.82–1.48)	0.508	1.000	0.71 (0.45–1.11)	0.508	1.000	0.99 (0.67–1.44)	0.944	1.000
rs1938496	66100915	G/A (0.169)	0.97 (0.67–1.39)	0.849	1.000	0.51 (0.30–0.85)	0.849	1.000	0.99 (0.63–1.58)	0.977	1.000
rs1805096	66102257	C/T (0.345)	0.93 (0.71–1.23)	0.623	1.000	1.34 (0.90–2.01)	0.623	1.000	0.86 (0.60–1.22)	0.385	0.996
rs1892534	66105944	G/A (0.347)	0.95 (0.72–1.25)	0.695	1.000	1.34 (0.90–2.01)	0.695	1.000	0.87 (0.62–1.24)	0.442	0.994
**CRC-specific survival**[c]											
rs12145690	65887013	A/C (0.444)	0.94 (0.64–1.37)	0.737	1.000	0.82 (0.45–1.52)	0.529	1.000	1.16 (0.68–1.97)	0.595	1.000
rs4655802	65888231	A/G (0.428)	1.05 (0.72–1.54)	0.801	1.000	1.32 (0.69–2.52)	0.407	0.999	0.88 (0.53–1.47)	0.632	1.000
rs9436297	65888854	T/C (0.132)	1.00 (0.59–1.72)	0.991	1.000	1.19 (0.44–3.22)	0.731	1.000	1.06 (0.54–2.06)	0.874	1.000
rs3790433	65894342	G/A (0.267)	1.35 (0.87–2.08)	0.184	0.934	1.02 (0.50–2.09)	0.967	1.000	2.12 (1.16–3.89)	0.015	0.252
rs7534511	65895130	G/A (0.311)	0.76 (0.54–1.08)	0.128	0.874	0.75 (0.41–1.39)	0.360	0.998	0.51 (0.30–0.85)	0.010	0.182
rs9436301	65895927	T/C (0.271)	1.47 (0.92–2.36)	0.106	0.837	1.29 (0.58–2.87)	0.529	1.000	2.05 (1.11–3.77)	0.021	0.322
rs1887285	65897747	T/C (0.090)	1.11 (0.57–2.18)	0.763	1.000	0.77 (0.25–2.37)	0.653	1.000	1.71 (0.72–4.04)	0.221	0.948
rs7602	65897951	G/A (0.219)	1.94 (1.11–3.39)	0.019	0.318	1.44 (0.61–3.39)	0.404	0.999	3.39 (1.55–7.40)	0.002	0.049
rs970467	65906762	G/A (0.136)	1.56 (0.82–2.96)	0.177	0.932	1.49 (0.55–4.10)	0.435	0.999	1.93 (0.80–4.64)	0.141	0.864

Table S3 Continued

SNP ID	Position[a]	Major/Minor allele (MAF)	All CRC			I/II			III/IV		
			HR (95% CI)[b]	*P*-value	P_{ACT}[d]	HR (95% CI)[b]	*P*-value	P_{ACT}[d]	HR (95% CI)[b]	*P*-value	P_{ACT}[d]
rs9436748	65911672	G/T (0.426)	0.90 (0.64–1.27)	0.547	1.000	1.09 (0.62–1.90)	0.765	1.000	0.82 (0.50–1.33)	0.417	0.993
rs6588147	65935494	A/G (0.338)	1.00 (0.69–1.45)	0.992	1.000	0.79 (0.40–1.56)	0.496	1.000	1.47 (0.89–2.45)	0.136	0.856
rs7513047	65950340	G/A (0.476)	1.08 (0.75–1.56)	0.678	1.000	1.40 (0.75–2.63)	0.291	0.991	0.94 (0.56–1.56)	0.804	1.000
rs17127673	65955725	A/G (0.115)	0.69 (0.34–1.41)	0.308	0.989	1.73 (0.60–5.01)	0.315	0.994	0.29 (0.10–0.86)	0.025	0.351
rs1327115	65966839	G/T (0.410)	1.20 (0.84–1.73)	0.315	0.989	1.21 (0.63–2.32)	0.561	1.000	1.36 (0.84–2.19)	0.206	0.939
rs11208659	65979280	T/C (0.095)	0.63 (0.36–1.12)	0.113	0.851	0.51 (0.20–1.27)	0.149	0.910	0.86 (0.37–2.00)	0.719	1.000
rs1475397	65983158	C/T (0.254)	0.93 (0.59–1.47)	0.761	1.000	1.72 (0.84–3.53)	0.140	0.900	0.38 (0.19–0.78)	0.009	0.162
rs1171278	65988146	C/T (0.160)	0.57 (0.31–1.08)	0.083	0.764	1.26 (0.49–3.24)	0.626	1.000	0.22 (0.09–0.55)	0.001	0.031
rs1751492	65992625	T/C (0.294)	0.96 (0.65–1.43)	0.856	1.000	1.35 (0.65–2.81)	0.417	0.999	0.56 (0.33–0.93)	0.026	0.338
rs6697315	65993556	T/C (0.368)	0.80 (0.56–1.16)	0.240	0.970	0.85 (0.44–1.64)	0.631	1.000	0.66 (0.42–1.04)	0.075	0.661
rs10749753	65998467	A/G (0.481)	1.00 (0.68–1.45)	0.970	1.000	0.82 (0.43–1.60)	0.568	1.000	1.12 (0.67–1.87)	0.679	1.000
rs12140910	66002660	A/G (0.074)	1.75 (0.97–3.16)	0.064	0.683	2.68 (1.10–6.55)	0.031	0.426	0.91 (0.36–2.28)	0.833	1.000
rs10889562	66030445	G/A (0.240)	0.86 (0.58–1.28)	0.456	0.999	0.79 (0.41–1.51)	0.468	1.000	0.79 (0.45–1.38)	0.403	0.993
rs1137100	66036441	A/G (0.265)	1.00 (0.67–1.51)	0.985	1.000	0.86 (0.42–1.75)	0.670	1.000	1.50 (0.87–2.60)	0.149	0.864
rs10493380	66046117	A/C (0.190)	0.90 (0.56–1.45)	0.665	1.000	0.72 (0.31–1.66)	0.437	0.999	0.83 (0.43–1.59)	0.575	1.000
rs6673591	66048389	G/A (0.480)	1.14 (0.80–1.63)	0.463	0.999	1.01 (0.54–1.88)	0.982	1.000	1.70 (1.05–2.75)	0.030	0.371
rs10749754	66054640	G/A (0.474)	0.95 (0.66–1.37)	0.787	1.000	1.05 (0.55–1.99)	0.885	1.000	0.57 (0.34–0.97)	0.038	0.440
rs1137101	66058513	A/G (0.471)	1.07 (0.75–1.53)	0.712	1.000	0.98 (0.52–1.85)	0.960	1.000	1.74 (1.03–2.95)	0.040	0.452
rs4655537	66058801	G/A (0.340)	0.89 (0.62–1.27)	0.515	1.000	0.67 (0.37–1.22)	0.187	0.947	1.20 (0.72–1.99)	0.483	0.996
rs12405556	66063117	G/T (0.265)	0.96 (0.64–1.44)	0.831	1.000	1.04 (0.52–2.06)	0.923	1.000	0.73 (0.42–1.27)	0.259	0.962
rs1938496	66100915	G/A (0.169)	0.75 (0.46–1.23)	0.256	0.977	0.77 (0.35–1.70)	0.521	1.000	0.76 (0.38–1.52)	0.439	0.994
rs1805096	66102257	C/T (0.345)	1.12 (0.76–2.65)	0.583	1.000	1.00 (0.52–1.90)	0.997	1.000	1.01 (0.60–1.70)	0.961	1.000
rs1892534	66105944	G/A (0.347)	1.12 (0.76–1.65)	0.583	1.000	1.00 (0.52–1.90)	0.997	1.000	1.01 (0.60–1.70)	0.961	1.000

CRC, colorectal cancer; HR, hazard ratio; MAF, minor allele frequency. [a]SNP locations were mapped according to the NCBI build 36 coordinates. [b]Hazard ratios were calculated with reference to the underlined allele. [c]Cox proportional hazard model adjusted for age at diagnosis, gender, race, stage at diagnosis, household income, reported screening procedure, marital status, family history, smoking status, alcohol consumption status, folate intake, MSI status, and BRAF mutation status, where applicable. [d]*P*-values were adjusted for multiple comparisons with a modification of P_{ACT} for correlated tests developed by Conneely and Boehnke[21].

Table S4 Associations between *LEP* SNPs and colorectal cancer overall survival, disease-free survival, and CRC-specific survival after stage at diagnosis stratification

SNP ID	Position[a]	Major/Minor allele (MAF)	All CRC			I/II			III/IV		
			HR (95% CI)[b]	*P*-value	P_{ACT}[d]	HR (95% CI)[b]	*P*-value	P_{ACT}[d]	HR (95% CI)[b]	*P*-value	P_{ACT}[d]
**Overall survival**[c]											
rs4731427	127884389	T/C (0.069)	0.88 (0.52–1.48)	0.625	1.000	2.37 (0.57–9.84)	0.235	0.970	0.70 (0.38–1.27)	0.243	1.000
rs11763517	127890062	T/C (0.483)	0.83 (0.64–1.09)	0.180	0.946	1.01 (0.66–1.55)	0.963	1.000	0.75 (0.52–1.09)	0.130	1.000
rs28954118	127895182	A/T (0.051)	1.33 (0.70–2.53)	0.390	0.998	2.88 (0.86–9.71)	0.087	0.773	0.63 (0.30–1.32)	0.220	1.000
**Disease-free survival**[c]											
rs4731427	127884389	T/C (0.069)	0.84 (0.52–1.33)	0.451	0.997	1.68 (0.52–5.41)	0.383	0.995	0.80 (0.47–1.37)	0.420	1.000
rs11763517	127890062	T/C (0.483)	0.66 (0.51–0.87)	0.003	0.058	0.82 (0.53–1.27)	0.380	0.997	0.63 (0.44–0.91)	0.014	1.000
rs28954118	127895182	A/T (0.051)	1.32 (0.72–2.43)	0.373	0.996	2.38 (0.72–7.87)	0.154	0.914	0.76 (0.39–1.49)	0.422	1.000
**CRC-specific survival**[c]											
rs4731427	127884389	T/C (0.069)	1.02 (0.44–2.36)	0.971	1.000	–	–	–	0.63 (0.26–1.56)	0.319	0.983
rs11763517	127890062	T/C (0.483)	1.34 (0.91–1.95)	0.136	0.886	0.96 (0.49–1.87)	0.893	1.000	1.81 (1.08–3.04)	0.025	0.345
rs28954118	127895182	A/T (0.051)	1.43 (0.52–3.97)	0.493	1.000	1.39 (0.29–6.61)	0.681	1.000	1.50 (0.32–7.12)	0.611	1.000

CRC, colorectal cancer; HR, hazard ratio; MAF, minor allele frequency. [a]SNP locations were mapped according to the NCBI build 36 coordinates. [b]Hazard ratios were calculated with reference to the underlined allele. [c]Cox proportional hazard model adjusted for age at diagnosis, gender, race, stage at diagnosis, household income, reported screening procedure, marital status, family history, smoking status, alcohol consumption status, folate intake, MSI status, and BRAF mutation status, where applicable. [d]*P*-values were adjusted for multiple comparisons with a modification of P_{ACT} for correlated tests developed by Conneely and Boehnke[21].
